# Pancreatic Involvement in the Course of Inflammatory Bowel Disease in Children—A Multi-Center Study

**DOI:** 10.3390/jcm12134174

**Published:** 2023-06-21

**Authors:** Urszula Daniluk, Paulina Krawiec, Elżbieta Pac-Kożuchowska, Łukasz Dembiński, Jan Stanisław Bukowski, Aleksandra Banaszkiewicz, Anna Woźniuk-Kaźmierczak, Elżbieta Czkwianianc, Jan Brylak, Jarosław Walkowiak, Agnieszka Borys-Iwanicka, Anna Kofla-Dłubacz, Tomasz Pytrus, Katarzyna Zdanowicz, Dariusz Marek Lebensztejn

**Affiliations:** 1 Department of Pediatrics, Gastroenterology, Hepatology, Nutrition, Allergology and Pulmonology, Medical University of Bialystok, 15-274 Bialystok, Poland; kazdanowicz@gmail.com (K.Z.); lebensztejn@hoga.pl (D.M.L.); 2 Department of Pediatrics and Gastroenterology, Medical University of Lublin, 20-059 Lublin, Poland; paulinakrawiec@umlub.pl (P.K.); elzbietapackozuchowska@umlub.pl (E.P.-K.); 3 Department of Pediatric Gastroenterology and Nutrition, Medical University of Warsaw, 02-091 Warsaw, Poland; lukaszdembinski@gmail.com (Ł.D.); jan.bukowski@wum.edu.pl (J.S.B.); aleksandra.banaszkiewicz@wum.edu.pl (A.B.); 4 Department of Gastroenterology, Allergology and Pediatrics, Polish Mother’s Memorial Hospital-Research Institute, 93-338 Lodz, Poland; ann.wozniuk@gmail.com (A.W.-K.); elzbieta.czkwianianc@iczmp.edu.pl (E.C.); 5 Department of Pediatric Gastroenterology and Metabolic Diseases, Poznan University of Medical Sciences, 60-572 Poznan, Poland; j.brylak@wp.pl (J.B.); jarwalk@ump.edu.pl (J.W.); 6 2nd Clinical Department of Pediatrics, Gastroenterology and Nutrition, Medical University of Wroclaw, 50-369 Wroclaw, Poland; agnieszka.borys-iwanicka@umw.edu.pl (A.B.-I.); anna.kofla-dlubacz@umw.edu.pl (A.K.-D.); tomasz.pytrus@orange.pl (T.P.)

**Keywords:** children, Crohn’s disease, hyperamylsemia, hyperlipasemia, inflammatory bowel disease, pancreatitis, ulcerative colitis

## Abstract

The coexistence of inflammatory bowel disease (IBD) with pancreatic pathology is rare in children. A retrospective analysis of data from 1538 children diagnosed with IBD in 2014–2021 was conducted to determine the frequency and causes of pancreatitis and asymptomatic hyperlipasemia (HL) or hyperamylasemia (HA) in this group of patients. Among the 176 children (11.4%) with pancreatic involvement (PI), acute pancreatitis (AP) was diagnosed in 77 children (43.8%), and HA or HL was observed in 88 children (50.0%). Only a few patients were diagnosed with autoimmune or chronic pancreatitis (6.2%). PI was observed at the time of the IBD diagnosis in 26.1% of the cases. A total of 54.5% of the patients had moderate to severe IBD, and 96% had colonic involvement at the time of diagnosis of PI. Idiopathic PI was the most common (57%), followed by drug-induced PI (37%) and azathioprine (AZA). In patients with AZA-induced AP, the successful introduction of 6-mercaptopurine (6-MP) to therapy was noted in 62.5% of the children. Our results suggest that routine monitoring of pancreatic enzymes in patients with IBD should be performed, especially after the initiation of the AZA treatment. The presence of transient HA/HL in IBD does not necessarily indicate pancreatic pathology.

## 1. Introduction

Inflammatory bowel disease (IBD) is a term used to describe diseases such as Crohn’s disease (CD), ulcerative colitis (UC) and IBD-unclassified (IBD-U). IBD is a chronic and relapsing–remitting inflammatory condition affecting the gastrointestinal tract, but it may also affect other organs and systems, such as joints, skin, heart, hepatobiliary tract or pancreas [1,2,3,4,5,6]. Extra-intestinal manifestations (EIMs) are observed in up to 50% of adult IBD patients [1,4,7]. However, the occurrence of EIM in children is less frequent and ranges from 6 to 28.2% [6,8,9].

In recent years, an increase in the incidence of pancreatitis in children has been observed [10]. The latest guidelines highlight the following pancreatitis: acute pancreatitis (AP), acute recurrent pancreatitis (ARP) and chronic pancreatitis (CP) [11,12]. The etiology of pancreatitis is complex and includes factors such as anatomical abnormalities, genetic predisposition, trauma, drugs, underlying systemic diseases and autoimmune pancreatitis (AIP) [11,13]. Pediatric AP is diagnosed if two of the three criteria are met as follows: (1) clinical symptoms (abdominal pain or vomiting), (2) serum amylase and/or lipase values at least three times higher than the normal threshold, and (3) radiological evidence of pancreatitis [12,14]. Due to its rarity, guidelines for the diagnosis of AIP in children have not yet been developed. The diagnosis is based on the criteria used in adults, considering symptoms, imaging and laboratory results, histology and response to therapy [12,14]. CP is diagnosed when at least one of the following three criteria is met: (1) irreversible structural changes in the pancreas and periods of consistent abdominal pain or increase in pancreatic enzymes; (2) irreversible structural changes in the pancreas with exocrine pancreatic insufficiency; (3) irreversible structural changes in the pancreas with endocrine pancreatic insufficiency [12].

The coexistence of pancreatic pathology and IBD has been increasingly described, but the cause-and-effect relationship between the two is yet to be established [6]. One of the possible explanations is the abnormal passage of pancreatic enzymes from the intestinal lumen to the blood due to the increased permeability of the inflamed mucosa in more extensive or active IBD [15]. On the other hand, the presence of pancreatic antibodies (PABs) or pancreatic-associated protein (PAP) in IBD patients suggests a close link between the pancreas and the gut, but its role is still unknown [16,17]. Pancreatic involvement (PI) coexisting with IBD includes AP, AIP, CP and asymptomatic hyperenzymemia (hyperamylasemia (HA) and/or hyperlipasemia (HL)) [6,18,19,20,21,22,23]. In children with IBD, pancreatitis is mainly caused by drugs used to treat IBD or systemic inflammation that can be considered EIM, or by a rare coexisting autoimmune mechanism or biliary disease [6,19,20,21,24,25,26,27]. The clinical significance of asymptomatic HA and HL remains unclear. However, it has been shown that one-quarter of the children with HA/HL develop acute pancreatitis within a one-year follow-up period [18]. Until now, only a few studies have explored the issue of exclusive HA/HL in patients with IBD [18,19,28,29].

The primary aim of our study was to analyze the frequency, type and cause of pancreatitis and HA/HL in children with IBD. The second goal was to evaluate whether PI is related to a different course of IBD.

## 2. Materials and Methods

Our retrospective study was conducted in six tertiary hospitals in Poland (Bialystok, Lublin, Lodz, Poznan, Warsaw and Wroclaw). Prior to the study, approval was obtained from the local Bioethics Committee at the Medical University of Bialystok (consent number: APK.002.508.2021), which waived the requirement for informed consent while maintaining patient anonymity due to the retrospective nature of this study. All methods were performed in accordance with the relevant guidelines and regulations and according to the principles laid down in the Declaration of Helsinki. Data were manually extracted from electronic medical records by the members of the research study. This analysis involved 1538 children diagnosed with IBD between 2014 and 2021 (641 CD and 897 UC) based on clinical, radiological, histological and endoscopic criteria according to the European Society for Pediatric Gastroenterology Hepatology and Nutrition (ESPGHAN) guidelines [30]. PI was defined as AP, HA/HL, AIP and CP. Diagnoses of AP, AIP and CP were established based on the International Study Group of Pediatric Pancreatitis: In Search for a Cure (INSPPIRE) criteria [12]. HA/HL was defined as an increase in pancreatic enzyme activity above the upper normal limit without accompanying symptoms or abnormal imaging results after excluding macroenzymemia.

Children diagnosed with IBD and with a history of the first episode of PI (n = 176) were included in the study. The clinical and laboratory data, including serum amylase and lipase activity, were collected from the medical records. CD activity was assessed using the Pediatric Crohn’s Disease Activity Index (PCDAI) and scored as remission (≤10), mild (11–27.5), moderate (>27.5–50) and severe (>50). The UC disease activity was estimated according to the Pediatric Ulcerative Colitis Index (PUCAI) and rated as inactive (<10 points), mild (10–34 points), moderate (35–64 points) or severe (≥65 points). The IBD phenotype was assessed based on the Paris classification, including age at the time of the diagnosis (A), disease location (L), disease behavior (B) in the case of CD or including extent (E) in the case of UC [31]. Data from radiological examinations (abdominal ultrasound, abdominal computed tomography or magnetic resonance cholangiopancreatography imaging) were evaluated. An analysis of IBD treatment was also performed. Drug-induced pancreatitis or HA/HL was diagnosed when its onset was related to the treatment introduction and the normalization of pancreatic enzyme activity after drug discontinuation was achieved. The severity of AP was classified as mild, moderately severe or severe according to the criteria of the North American Society for Pediatric Gastroenterology, Hepatology and Nutrition (NASPGHAN) Pancreas Committee [32]. Mild AP is recognized if it is not associated with any organ failure or local or systemic complications and generally resolves within the first week of onset. Moderately severe AP is defined as AP with transient organ dysfunction lasting less than 48 h or local or systemic complications are present. Severe AP is diagnosed when organ failure persists for more than 48 h [32].

Descriptive data were analyzed using the Statistica 13.3 software. Categorical data were expressed as frequencies and percentages, and continuous variables are expressed as medians (min-max). The differences between the groups were tested for significance using the Mann–Whitney U test for continuous variables, and the chi-square test with Yates’ correction for categorical variables. Statistical analysis was omitted in small groups of less than 5 patients. Statistical significance was noticed with *p* value < 0.05.

## 3. Results

### 3.1. PI in Course of IBD in Children

Among 1538 children diagnosed with IBD in 2014–2021, 176 children (11.4%) were additionally diagnosed with pancreatitis or HA/HL, including 46 children (26.1%) diagnosed with both IBD and PI at the same time. The demographic and clinical details collected at the time of the PI diagnosis are presented in Table 1.

In the studied group, the median age of the children was 14 years, and there was almost the same percentage of boys and girls, as well as CD and UC cases (Table 1). In our study group, there were no children with IBD-U. 54.5% (96/176) of the children had moderate to severe bowel disease activity at the time PI was recorded. The majority of the children with CD (91%, 71/78) had colonic involvement. Half of the patients with PI were diagnosed with pancreatitis, with AP as the most common cause, whereas the other half were diagnosed with HA/HL. The cause of AP or HA/HL was considered idiopathic in 57.0% (94/176) of the cases, whereas drug-induced AP or HA/HL was the second most frequently reported cause (36.9%, 61/165). Azathioprine (AZA) was found to be most commonly responsible for PI (24.8%, 41/165), followed by 5-aminosalicylic acid (5-ASA) (10.9%, 18/165). Among the other causes of AP or HA/HL, bowel surgery (n = 5), cholelithiasis (n = 2) and pediatric inflammatory multisystem syndrome temporally associated with severe acute respiratory syndrome coronavirus 2 (SARS-CoV-2) (MIS-C /PIMS-TS) (n = 3) were reported. Among other drugs, valproic acid (n = 1) and cyclosporine (n = 1) were registered. There was no case of PI induced by anti-TNFalfa monoclonal antibody in the study group.

When comparing patients with CD to patients with UC, no differences were found in the incidence of PI in terms of gender, age, and IBD activity, except that severe IBD activity was more frequently found in patients with UC (26.5%, 26/98 vs. 11.5%, 9/78; *p* = 0.02), similar to the diagnosis of HA/HL in the CD group (60.3%, 47/78 vs. 41.8%, 41/98; *p* = 0.02) (Table 1).

### 3.2. AP and HA/HL in Course of IBD in Children

Since the most common pathologies of the pancreas in children with IBD are AP and HA/HL, we decided to compare these two groups of patients (Table 2).

We did not include the AIP and CP groups in the statistical analysis due to the small size of these groups, so data comparisons between the four groups were not made. Considering the age and gender, no differences were found between the AP and HA/HL groups. Among the children with AP, there were significantly more patients with UC (63.3%) compared to the HA/HL group, which had more patients with CD (53.4%). However, an active disease, assessed from mild to severe, was reported more frequently in the AP group compared to the HA/HL group (92.2%, 71/77 vs. 75.0%, 66/88; *p* = 0.006). The pancreatic enzyme activity levels were significantly higher in the AP group than in the HA/HL group. The majority of the AP cases were mild and none of the children had severe AP (Table 2). The percentages of children who presented with either AP or HA/HL at the time of the IBD diagnosis were comparable (19.5% and 26.1%, respectively). Interestingly, when comparing the etiology of PI, we noted that the incidence of an idiopathic cause was significantly higher in the HA/HL group than in the AP group, where drug-induced AP predominated (Table 2). Among the medications responsible for AP or HA/HL, we found AZA as the most frequently noted, followed by 5-ASA (Table 1 and Table 2).

Due to the finding that AZA and 5-ASA were the most common causes of AP and HA/HL, we compared both pancreatic pathologies according to the treatment used (Table S1). Both drugs induced AP more frequently than HA/HL did (82.9% and 83.3% vs. 17.1% and 16.7%). Comparing AP induced by AZA and 5-ASA, higher values of PUCAI (*p* = 0.01) were found in patients treated with 5-ASA. As presented in Table S1, the severity of AP and the clinical activity of IBD did not differ significantly between children with AZA-induced pancreatitis and those with 5-ASA-induced AP. The median delay from the treatment initiation to the appearance of AP symptoms was 14 days for AZA-induced pancreatitis and 6.5 days for 5-ASA-induced AP (Table S1). Each patient with drug-induced AP had treatment modifications. In eight children with AZA-induced pancreatitis, 6-mercaptopurine (6-MP), the active metabolite of AZA, was chosen to replace AZA. Successful introduction of 6-MP to therapy was observed in 62.5% (5/8) of the children (two patients with CD and three patients with UC). The resolution of AP symptoms after discontinuation of AZA was observed within 1–10 days. Pancreatic enzymes were normalized after 1–115 days for lipase and 1–65 days for amylase. In four cases of 5-ASA-induced pancreatitis, the reintroduction of 5-ASA from another manufacturer was successful in 75% (3/4) of the patients. The time between the withdrawal of 5-ASA and the resolution of pancreatitis symptoms varied between 2 and 14 days, and the median time of enzyme normalization was 27 days for lipase and 13.5 days for amylase (Table S1).

### 3.3. PI in Relation to the Time of IBD Diagnosis

Finally, we decided to investigate whether there were any differences between patients diagnosed with PI at the time of diagnosis of IBD and patients with PI diagnosed at a later time (Table 3). It should be noted that children with newly diagnosed PI and IBD were not treated before.

There were no significant differences in age, gender, IBD type and lipase activity between the two groups. As shown in Table 3, patients who were simultaneously diagnosed with PI and IBD had higher PUCAI and PCDAI values, as well as higher amylase activity compared to those who were diagnosed with PI after the diagnosis of IBD. AP was more frequently diagnosed later when children were already treated for IBD (*p* = 0.026).

## 4. Discussion

To the best of our knowledge, this is the first multicenter study in Poland to report pancreatic disorders in IBD children and one of the few studies that refers to asymptomatic HA/HL as well as pancreatitis. From 2014 to 2021, we identified 1538 children with IBD, 11.4% of whom had a concomitant diagnosis of pancreatitis or HA/HL. Out of the 176 children with IBD and pancreatic involvement, 50% had asymptomatic HA/HL, 43.8% had acute pancreatitis and 6.2% had autoimmune or chronic pancreatitis. We observed that pancreatic involvement was associated with colonic disease and clinically active IBD. In the majority of the cases, the diagnosis of pancreatic involvement followed the recognition of IBD.

Pancreatitis is reported in up to 9.6% of the children with IBD and with a similar frequency in children with CD and UC disease [8,9,18,33]. The spectrum of PI in the course of IBD in the pediatric population according to published data includes AP as the most frequently reported, whereas the isolated HA/HL has been reported less frequently [8,9,18,19,21,24,25,26,27,28,29,33,34,35,36,37,38]. In our study, 11.4% of the children had comorbid IBD and PI, with both isolated HA/HL and AP being equally frequent. Moreover, 26.1% of the cases were diagnosed with both IBD and PI simultaneously, compared to 18.5% of the cases in the study by Martinelli et al. [18]. In a multicenter study from Israel, idiopathic AP preceded the diagnosis of IBD by an average of 24 weeks in 2.17% of the children [21]. In our study, AP was diagnosed in 29.2% of the newly diagnosed and untreated children with IBD. This high percentage may be because abdominal pain, which is one of the three criteria for the diagnosis of acute pancreatitis, is also a symptom of IBD. Moreover, in mild AP, pancreatic imaging changes are not always found. Based on the literature, both AIP and CP have been reported in only a few children with IBD, as in our results [20,22,23,39,40,41,42,43]. Interestingly, we observed that AIP was recognized more frequently at the onset of IBD in treatment-naïve children than in patients already treated for IBD. However, since the number of children with AIP in our study group was relatively low, further studies should investigate this issue. The novelty of our observations is the pancreatic complications following infection with the SARS-CoV-2 virus. Among the reported etiologies of AP and HA/HL, the majority of cases were related to the drugs used. Less often, it was the idiopathic form or the result of bile duct disease (three cases) or pancreas divisum (one case) [18,19,21,24,25,26,27,28,29,34,35,36,37,38,44,45,46,47,48,49,50,51,52,53,54,55,56,57]. In contrast, in our study, the cause of AP or HA/HL was considered idiopathic in 57% of the cases, whereas drug-induced AP or HA/HL was the second most frequently reported cause (37%). Interestingly, we noticed that the incidence of an idiopathic cause in the HA/HL group was significantly higher than that in the AP group, which may be justified by the fact that drugs are a known factor of AP, while HA/HL itself has an unknown/idiopathic etiology. The active IBD, as well as the female gender, were listed among the risk factors for AP [18]. The majority of our patients manifested active IBD, and 96.0% of the children had colonic disease, similar to the findings of Martinelli et al. [18]. However, no gender relationship was observed in our study. The reports showed that PI in children is equally common in both types of IBD, with AIP being more common in UC [6,18,20]. In addition, our results revealed more cases of isolated HA/HL in CD patients. It can be speculated that this is related to the nature of CD itself, in which the inflammation may affect the entire thickness of the gastrointestinal tract wall, thus causing its increased permeability to pancreatic enzymes compared to UC, where the changes are mainly superficial. There is little data on the severity of AP; only three studies found it to be mild, which we also observed in most cases in our study [18,21,24]. In the Martinelli et al. study, 25% of the children with an idiopathic HA/HL developed AP after 6 months of observation, and interestingly, only 68.7% of the children achieved complete pancreatic enzyme normalization after 12 months of observation [18]. The involvement of the pancreas in the course of IBD may be considered an EIM or a separate co-morbid disease, but it can also be a side effect of IBD therapy; therefore, a differential diagnosis should always be performed. It is still unclear whether pancreatic enzyme activity should be monitored during follow-up visits in the absence of clinical signs of pancreatic dysfunction. On the other hand, when we consider the available data on idiopathic pancreatic pathology of IBD in adults, 38% of patients with CD have pancreatic fibrosis, and 53% of patients with UC have chronic interstitial pancreatitis in autopsy tests [58]. Among asymptomatic patients with IBD, 11% have elevated levels of pancreatic enzymes, which is three times above the upper limits of normal (ULN) amylase, and 14% of patients have elevated lipase and amylase levels two times above ULN [59,60]. Thus, based on these reports and our results, the presence of HA/HL in the course of IBD, especially in the exacerbation phase, does not necessarily indicate a pancreatic pathology. On the one hand, this high prevalence of increased enzyme activity prompts us to monitor the result, but if it is transient and decreases with the cessation of inflammation, it is not associated with a change in the therapeutic management of IBD. In contrast, macroamylasemia as the cause of hyperamylasemia has only been reported in a single case report of a child with IBD [28].

As already mentioned, in our study, drugs were reported as the second most common cause of PI (37%) in children with IBD, with almost equal percentages in CD and UC. Medications were more likely to cause AP than HA/HL (28.4% vs. 6.3%), and in the majority of cases, the course of drug-induced AP was mild. In the Bai et al. study on children with drug-induced pancreatitis, nearly 10% had CD and 9% had UC [38]. On the other hand, among children with IBD treated with thiopurines, up to 5% of patients developed AP according to the available literature [19,26,27,34,35,36,37]. In our study, AZA-induced PI was diagnosed in 24.8% of the cases, mainly in the CD group, followed by 5-ASA (10.9%). Based on the available literature, other drugs and even biologics (e.g., vedolizumab) can also induce AP in children with IBD [24,25,48,49,50,51,52,53,54]. There was no case of PI induced by the anti-TNFalfa monoclonal antibodies in our study group. The mechanisms of action of the drugs leading to pancreatitis include hypersensitivity reactions, genetic predisposition, direct toxic effects, immune reactions or idiosyncratic reactions, i.e., abnormal biochemical transformation of the drug [6]. Azathioprine use has been associated with the development of AP or HA/HL in children with IBD within 90 days of starting treatment, most often in the first month [19,26]. In our study, the median time to AP following IBD treatment initiation was 14 days for AZA and 6.5 days for 5-ASA. According to the definition of drug-induced AP, symptoms must resolve after drug discontinuation (between 2 weeks and 2 months) [6]. The resolution of AP symptoms after discontinuation of AZA was observed within 1–10 days and after 5-ASA 2–14 days. However, pancreatic enzymes normalized for a longer period of time, even over five months in our observations. No relationship has been found between the development of pancreatitis and the dose of thiopurines, the level of their metabolites or the genotype of thiopurine methyltransferase (TMPT), which is known to be responsible for bone marrow toxicity [19,27,34,44,47]. Successful introduction of 6MP after AZA-induced AP in children with CD has been reported, but not in all cases [27,44,45,46]. In our study, we also observed the successful introduction of 6-MP to therapy in 62.5% of the children (two patients with CD and three patients with UC). Currently, there are no recommendations for the management of drug-induced HA/HL. However, taking into consideration the possibility of the development of pancreatic complications, such guidelines should be established. Further long-term studies should be performed to assess pancreatic function in children diagnosed with drug-induced HA/HL.

The limitation of our study is the retrospective assessment and short-term follow-up after one PI episode during the course of IBD. The strength of our study is the large number of children with IBD included in the study.

## 5. Conclusions

In conclusion, the manifestation of pancreatic disorders in the course of IBD in children varies from an asymptomatic elevation of pancreatic enzymes to AP. Pancreatic involvement, mainly idiopathic and drug-induced AP or HA/HL, was found in quite a large percentage of children with IBD in our study. Interestingly, children with colonic involvement were predominant in the study group. The PI was related to the activity of IBD, and it coexisted with the diagnosis of IBD in some pediatric patients. The course of AP, despite the etiology, was mild in most cases. The results of our study highlight the need for routine monitoring of pancreatic enzyme activity in patients with IBD, particularly after starting treatment with azathioprine or 5-aminosalicylates. However, the presence of HA/HL in the course of IBD, especially in the exacerbation phase, does not necessarily indicate pancreatic pathology. If it is transient and decreases with the cessation of inflammation, it is not associated with a change in the therapeutic management of IBD. Further long-term studies on pancreatic function in children diagnosed with drug-induced HA/HL are needed in order to establish appropriate management recommendations.

## Figures and Tables

**Table 1:** Demographic data and clinical manifestation in CD and UC groups that were collected at the time of the diagnosis of PI.

Characteristics	CD n = 78 (44.3%)	UC n = 98 (55.7%)	*p* *	Total IBD n = 176
Age (median years, range)	14.0 (1–18)	13.0 (2–17)	0.65	14.0 (1–18)
Gender (n, %)				
Males	48 (61.5)	46 (46.9)	0.05	94 (53.4)
IBD activity (n, %)				
Remission	16 (20.5)	13 (13.3)	0.19	29 (16.5)
Mild	22 (28.2)	29 (29.6)	0.84	51 (29.0)
Moderate	31 (39.7)	30 (30.6)	0.21	61 (34.7)
Severe	9 (11.5)	26 (26.5)	**0.02**	35 (19.9)
PUCAI (median, range)	NA	40.0 (0–85)	NA	40.0 (0–85)
PCDAI (median, range)	30.0 (0–70)	NA	NA	30.0 (0–70)
Paris classification (n)				
Location-L1/L2/L3/L4	6/26/45/31		NA	NA
Behavior-B1/B2/B3	69/6/3		NA	NA
Extent-E1/E2/E3/E4		7/22/17/52	NA	NA
IBD treatment during diagnosis of PI (n, %)				
Steroids	22 (28.2)	36 (36.7)	0.23	58 (33.0)
5-ASA	47 (60.3)	66 (67.3)	0.33	113 (64.2)
AZA	51 (65.4)	31 (31.6)	**<0.001**	82 (46.6)
TNF-alpha inhibitors	11 (14.1)	11 (11.2)	0.56	22 (12.5)
Cyclosporine	0 (0.0)	4 (4.1)	NA	4 (2.3)
MTX	2 (2.6)	1 (1.0)	NA	3 (1.7)
PI (n, %):				
AP	28 (35.9)	49 (50.0)	0.06	77 (43.8)
HA/HL	47 (60.3)	41 (41.8)	**0.02**	88 (50.0)
AIP	3 (3.9)	6 (6.1)	NA	9 (5.1)
CP	0 (0.0)	2 (2.0)	NA	2 (1.1)
Etiology of AP and HA/HL (n, %):	n = 75	n = 90		n = 165
AZA	26 (34.7)	15 (16.7)	**0.001**	41 (24.8)
5-ASA	2 (2.7)	16 (17.8)	**0.004**	18 (10.9)
Other drugs	0 (0.0)	2 (2.2)	NA	2 (1.2)
Other	6 (8.0)	4 (4.4)	NA	10 (6.1)
Idiopathic	41 (54.7)	53 (58.9)	0.58	94 (57.0)
PI at IBD onset (n, %)	15 (19.2)	31 (31.6)	0.06	46 (26.1)

* CD vs. UC. CD—Crohn’s disease; UC—ulcerative colitis; IBD—inflammatory bowel disease; PCDAI—Pediatric Crohn’s Disease Activity Index; PUCAI—Pediatric Ulcerative Colitis Activity Index; 5-ASA—5-aminosalicylic acid. Location: L1—ileum only; L2—colon only; L3—ileum and colon; L—upper gastrointestinal tract. Behavior: B1—B2/B3. Extent: E1—proctosigmoiditis; E2—left-sided colitis; E3—extensive colitis; E4—pancolitis; PI—pancreatic involvement; AZA—azathioprine; MTX—methotrexate; TNF-alpha—Tumor Necrosis Factor-alpha; AP—acute pancreatitis; HA/HL—hyperamylasemia/hyperlipasemia; AIP—autoimmune pancreatitis; CP—chronic pancreatitis. NA—not applicable.

**Table 2:** The characteristics of patients with AP, HA/HL, AIP and CP.

Characteristics	AP n = 77 (43.8%)	HA/HL n = 88 (50.0%)	AIP n = 9 (5.1%)	CP n = 2 (1.1%)	* p * * AP vs. HA/HL
Age (median, range)	13.0 (2–17)	14.0 (1–17)	12.0 (4–17)	14.0 (12–16)	0.56
Males (n, %)	42 (54.5)	47 (53.4)	4 (44.4)	1 (50.0)	0.88
CD (n, %)	28 (36.4)	47 (53.4)	3 (33.3)	0 (0.0)	** 0.04 **
UC (n, %)	49 (63.6)	41 (46.6)	6 (66.7)	2 (100.0)	** 0.04 **
IBD activity (n, %)					
Remission	6 (7.8)	22 (25.0)	1 (11.1)	0 (0.0)	**0.006**
Mild	26 (33.8)	25 (28.4)	0 (0.0)	0 (0.0)	0.46
Moderate	35 (45.5)	21 (23.9)	4 (44.4)	1 (50.0)	**0.006**
Severe	10 (13.0)	20 (22.7)	4 (44.4)	1 (50.0)	0.10
PUCAI (median, range)	40.0 (0–80)	35.0 (0–85)	52.5 (0–70)	55.0 (40–70)	0.73
PCDAI (median, range)	35.0 (5–75)	25.0 (0–70)	45.0 (42–52.5)	NA	0.11
Colonic involvement (n, %)	77 (100)	81 (92.0)	9 (100)	2 (100)	NA
Severity of AP (n, %)					
Mild	72 (93.5)	NA	NA	NA	NA
Moderate	5 (6.5)	NA	NA	NA	NA
Severe	0 (0.0)	NA	NA	NA	NA
PI at IBD onset (n, %)	15 (19.5)	23 (26.1)	6 (66.7)	2 (100.0)	0.31
Symptoms (n, %):					
Epigastric pain	68 (88.3)	0 (0.0)	4 (44.4)	0 (0.0)	NA
Nausea/Vomiting	26 (33.8)	0 (0.0)	0 (0.0)	0 (0.0)	NA
Amylase IU/L (median, range)	178 (30–1990)	114 (33–932)	148 (62–413)	-	** <0.001 **
Lipase IU/L, median (range)	638 (180–11,108)	245 (35–5010)	779 (229–5689)	381.5 (230–533)	** <0.001 **
Pancreatic abnormalities in imaging studies (n, %)	33 (42.9)	0 (0.0)	6 (66.7)	2 (100.0)	NA
Etiology of AP and HA/HL (n, %)					
Drugs:	50 (64.9)	11 (12.5)	NA	NA	**<0.0001**
AZA	34 (68.0)	7 (63.6)	NA	NA	0.78
5-ASA	15 (30.0)	3 (27.3)	NA	NA	NA
Other drugs	1 (2.0)	1 (9.1)	NA	NA	NA
Other	5 (6.5)	5 (5.7)	NA	NA	NA
Idiopathic	22 (28.6)	72 (81.8)	NA	NA	**<0.0001**

* AP vs. HA/HL. AP—acute pancreatitis; HA/HL—hyperamylasemia/hyperlipasemia; IBD—inflammatory bowel disease; CD—Crohn’s disease; UC—ulcerative colitis; PCDAI—Pediatric Crohn’s Disease Activity Index; PUCAI—Pediatric Ulcerative Colitis Activity Index; PI—pancreatic involvement; 5-ASA—5-aminosalicylic acid; AZA—azathioprine; NA—not applicable.

**Table 3:** Comparison of patients with IBD at the time of the diagnosis of PI.

	PI at the Time of IBD Diagnosis (n = 48, 27.3%)	PI after IBD Diagnosis (n = 128, 72.7%)	* p *
Age (median, range)	14.0 (2–17)	13.5 (1–17)	0.73
Gender (n, %) Males	26 (54.2)	68 (53.1)	0.96
CD (n, %)	15 (31.3)	61 (47.7)	0.07
UC (n, %)	33 (68.8)	67 (52.3)	0.07
IBD activity (n, %)			
Remission	0 (0.0)	29 (22.7)	NA
Mild	11 (22.9)	40 (31.3)	0.37
Moderate	23 (47.9)	38 (29.7)	**0.04**
Severe	14 (29.2)	21 (16.4)	0.09
PUCAI (median, range)	45.0 (10–85)	30.0 (0–75)	** 0.03 **
PCDAI (median, range)	38.5 (0–65)	25.0 (0–75)	** 0.002 **
Colon involvement (n, %)	44 (91.7)	126 (98.4)	0.08
Amylase, IU/L (median, range)	148 (34–1990)	109 (33–501)	** 0.008 **
Lipase, IU/L (median, range)	450 (60–11,180)	430 (35–5689)	0.66
PI (n, %):			
AP	14 (29.2)	63 (49.2)	** 0.026 **
Severity of AP:			
Mild	12 (85.7)	60 (95.2)	0.47
Moderately severe	2 (14.3)	3 (4.8)	NA
Severe	0 (0.0)	0 (0.0)	NA
HA/HL	26 (54.2)	62 (48.4)	0.61
AIP	6 (12.5)	3 (2.3)	NA
CP	2 (4.2)	0 (0.0)	NA

IBD—inflammatory bowel disease, CD—Crohn’s disease, UC—ulcerative colitis, PUCAI—Pediatric Ulcerative Colitis Activity Index, PCDAI—Pediatric Crohn’s Disease Activity Index, PI—pancreatic involvement, AP—acute pancreatitis, HA/HL—hyperamylasemia/hyperlipasemia, AIP—autoimmune pancreatitis, CP—chronic pancreatitis, and NA—not applicable.

## Data Availability

The datasets generated and analyzed during the current study are available from the corresponding author upon reasonable request.

## Supplementary Materials

The following supporting information can be downloaded at: https://www.mdpi.com/article/10.3390/jcm12134174/s1. Table S1: Characteristics of patients with AP and HA/HL caused by AZA or 5-ASA treatments.
