# Supplementary material for: Pancreatic Involvement in the Course of Inflammatory Bowel Disease in Children—A Multi-Center Study
Source: J Clin Med. 2023 Jun 21;12(13):4174. doi: 10.3390/jcm12134174 (PMC10342734; doi:10.3390/jcm12134174)
Supplement: Supplementary file 1 [file jcm-12-04174-s001.zip › jcm-2456583-supplementary.pdf]

Table S1. Characteristics of patients with AP and HA/HL caused by AZA or 5-ASA treatments.

Characteristics	AZA n=41		5-ASA n=18		p* AZA AP vs 5-ASA AP
PI	AP (n=34, 82.9%)	HA/HL (n=7, 17.1%)	AP (n=15, 83.3%)	HA/HL (n=3, 16.7%)	0.96
Age (median, range)	12.5 (3-17)	12.0 (7-16.5)	14.5 (8-17)	14.0 (13-15)	0.38
Males (n, %)	18 (52.9)	7 (100.0)	4 (26.7)	1 (33.3)	NA
CD (n, %)	20 (58.8)	6 (85.7)	2 (13.3)	0 (0.0)	NA
UC (n, %)	14 (41.2)	1 (14.3)	13 (86.7)	3 (100)	**0.01**
IBD activity (n, %)					
Remission	1 (2.9)	0 (0.0)	1 (6.7)	0 (0.0)	NA
Mild	17 (50.0)	4 (57.1)	3 (20.0)	0 (0.0)	NA
Moderate	14 (41.2)	2 (28.6)	7 (46.7)	1 (33.3)	0.95
Severe	2 (5.8)	1 (14.3)	4 (26.7)	2 (66.7)	NA
PUCAI (median, range)	22.5 (0-65)	70.0 (70-70)	55.0 (10-70)	65.0 (30-75)	**0.01**
PCDAI (median, range)	31.25 (5-52.5)	21.25 (12.5-40)	25 (7.5-25)	NA	0.23
Severity of AP (n, %)					
Mild	32 (94.1)	NA	14 (93.3)	NA	0.19
Moderately sever	2 (5.9)	NA	1 (6.7)	NA	NA
Sever	0 (0.0	NA	0 (0.0)	NA	NA
Symptoms (n, %):					
Abdominal pain	30 (88.2)	NA	13 (86.7)	NA	0.28
Vomiting	13 (38.2)	NA	3 (20.0)	NA	NA
Amylase, IU/L (median, range)	178 (34-1990)	175 (118-932)	221.5 (76-698)	79 (45-113)	0.35
Lipase, IU/L (median, range)	638 (180-11108)	245 (35-5010)	779 (229-5689)	110 (93-311)	0.08
Pancreatic abnormalities in imaging studies (n, %)	14 (41.2)	NA	8 (53.3)	NA	0.51

Number of days from the onset of treatment to the presence of AP/HA/HL (median, range)	14.0 (1-30)	30.0 (25-15)	6.5 (4-18)	5.0 (3-6)	NA
Number of days (median, range) to:					
- resolution of symptoms	3.0 (1-10)	NA	4.0 (2-14)	NA	NA
- lipase normalization	10.0 (1-115)	19.0 (7-85)	27.0 (2-150)	3.0 (2-3)	NA
- amylase normalization	4.5 (1-65)	5.5 (0-37)	13.5 (1-377)	NA	NA

p* - AZA-AP vs 5-ASA-AP, PI – pancreatic involvement, 5-ASA – 5-aminosalicylic acid, AP – acute pancreatitis, AZA – azathioprine, CD – Crohn's disease, HA/HL – hyperamylasemia/hyperlipasemia, IBD – inflammatory bowel disease, PCDAI – pediatric Crohn's disease activity index, PUCAI – Pediatric Ulcerative Colitis Activity Index, UC – ulcerative colitis, NA – not applicable
